# Glances at the Insane in Other Lands

**Published:** 1888-12-01

**Authors:** 


### Glances at the Insane in Other Lands.

By a Medical Superintendent.

### I.—NEW SOUTH WALES.

Dr. Manning, the Inspector-General, whose report we are about to consider, is well known in many English asylums, not alone from personal visits to them, but as being the author of one of the very best Blue Books ever issued on English asylums. In the document now under notice he gratefully mentions his second visit to the mother country, and names those asylums which he once more honoured with a call. We note his being at Cane Hill, Gloucester, Menstone, Berry Wood, Barnwood House, Morningside, Hanwell, Darenth, etc., etc.

The Colony of New South Wales now contains 2,872 registered lunatics, and during the last year they increased by 104. The total population of the colony is a little over a million, and the proportion of the insane was 1 in 369, being at the rate of 2·71 per 1,000, so that the ratio compares favourably with that of England, which is 2·86 per 1,000. The percentage of recoveries calculated in the usual way on the admissions is 40·22, which closely follows that pertaining in English asylums; while the deaths are lower than in England, standing at 6·79 per cent. on the average number resident. Nor is this low rate exceptional, for we find it stated that during the last quinquennial period the deaths were only 6·52 per cent.

The total number of cases admitted to the asylums of New South Wales during 1887 was 532, and Table 3 of the document tells us that in not fewer than 81 of these the insanity was brought on by drink. Hereditary influence accounts for 41. These two causes, although occupying the two first places, as with us, change their relative positions when compared with English asylums. There were 185 deaths, general paralysis being the cause in 18, and other brain diseases in 29. There were 11 deaths from inflammatory lung mischief, but only 8 from consumption. It would be most interesting to know how many of these eight were English-born. There was only one suicide. Turning to the escapes, we find that 34 patients managed to get away; but only 4 remained out long enough to obtain their formal discharge, and one of these returned voluntarily to the asylum at Paramatta.

As might be expected, the cost per head per week is higher in the Colony than in England, but it cannot be called excessive. It is a fraction under 12*s*. New South Wales possesses a Reception House for the Insane, with the management and constitution of which we should like to be further acquainted. It would seem that 555 patients were admitted to it during the year. Of these 271 were sent to asylums, 160 to police courts and then discharged as sane, 85 were sent to police courts, certified and returned as insane, 28 were discharged of sound mind, 7 died, and 11 remained at the close of the year.

We note with much satisfaction that the institutions are known as hospitals, and not as asylums. That at Gladsville contains 778 patients, being 63 more than it is intended to accommodate. Typhoid fever broke out, and altogether six persons were affected by the disease, two of them dying of it. The cause of the outbreak could not be ascertained. Several minor improvements were carried out; but beyond renting a house in the neighbourhood nothing seems to have been done to relieve the overcrowding.

At Paramatta 969 patients were resident at the close of the year. It, too, would seem to be overcrowded, and we learn that there are no rooms in the hospital for the assistant medical officer, so that he has to sleep elsewhere, a condition of things which can hardly be understood in England, and which cannot conduce to his own comfort or to that of the medical superintendent. The medical superintendent deplores the deficiency of single-bedded rooms, and justly remarks on the increased risk of accidents which this deficiency entails. We are informed that several of the staff of this hospital retired under the provisions of the Civil Service Pension Act, a statement which shows that the new country is in advance of the old in at least one very important particular.

From the report of the Callan Park Hospital, we learn that there were 668 patients resident on the last day of December. The amusement of the patients is provided for by picnics, dances, cricket matches, and concerts. It appears to have been customary for some of the patients to take trips on the river in a steam launch, a form of recreation which, so far as we know, has not yet been naturalised in England in any of our county asylums.

The criminal asylum contains 62 patients. It is situated at Paramatta, and is apparently under the same management as the main hospital in that district.

After visiting the Tower of London, poor Artemus Ward wept because his country possessed no such "sweet boon" as a "tower." New South Wales cannot exactly weep the absence of such "sweet boons" as private asylums, but it almost might, for it possesses only one. This is situated near Cook's River, and it contains 104 patients—23 of these being men, and 83 women.

